# Fabrication and Optimization of Vertically Aligned ZnO Nanorod Array-Based UV Photodetectors via Selective Hydrothermal Synthesis

**DOI:** 10.1186/s11671-015-1032-y

**Published:** 2015-08-12

**Authors:** Yeong Hwan Ko, Goli Nagaraju, Jae Su Yu

**Affiliations:** Department of Electronics and Radio Engineering, Institute for Laser Engineering, Kyung Hee University, 1 Seocheon-dong, Giheung-gu, Yongin-si, Gyeonggi-do 446-701 Republic of Korea

**Keywords:** Zinc oxide nanorod arrays, Ultraviolet photodetectors, Hydrothermal method

## Abstract

Vertically aligned ZnO nanorod array (NRA)-based ultraviolet (UV) photodetectors (PDs) were successfully fabricated and optimized via a facile hydrothermal process. Using a shadow mask technique, the thin ZnO seed layer was deposited between the patterned Au/Ti electrodes to bridge the electrodes. Thus, both the Au electrodes could be connected by the ZnO seed layer. As the sample was immersed into growth solution and heated at 90 °C, the ZnO NRAs were crystallized and vertically grown on the ZnO seed layer, thus creating a metal-semiconductor-metal PD structure. To investigate the size effect of ZnO NRAs on photocurrent, the PDs were readily prepared with different concentrations of growth solution. For the ZnO NRAs grown at 25 mM of concentration, the PD with 10 μm of channel width (i.e., gap distance between two electrodes) exhibited a high photocurrent of 1.91 × 10^−4^ A at an applied bias of 10 V under 365 nm of UV light illumination. The PD was optimized by adjusting the channel width. For 15 μm of channel width, a relatively high photocurrent on-off ratio of 37.4 and good current transient characteristics were observed at the same applied bias. These results are expected to be useful for cost-effective and practical UV PD applications.

## Background

Zinc oxide (ZnO) nanorod array (NRA)-based photodetectors (PDs) are alternative ultraviolet (UV) sensors because they have several advantages such as wide direct band gap (3.37 eV) and large surface area [1–3]. Compared to conventional ZnO thin-film UV PDs, it has been revealed that the nanostructured ZnO UV PDs can offer higher photocurrents [4–6]. In particular, one-dimensional (1D) ZnO nanostructures such as nanowires and nanorods have exhibited many advantages including large surface to volume ratio, high quantum efficiency, and direct pathway of charge transport [7, 8]. Furthermore, the reduced dimensionality enhances carrier lifetime and photoresponse properties [9]. Currently, the most popular approach for the fabrication is based on a metal-semiconductor-metal (MSM) structure by direct growth of lateral ZnO NRAs because the MSM photodetector is simple for easy fabrication and compatible with various semiconductor nanomaterials [10–13]. However, there are still technical difficulties for practical fabrication processes of PDs. In order to grow ZnO NRAs as an active channel between electrodes, the ZnO seed layer should be selectively etched after photolithography process. Although the ZnO nanorods are easily grown by chemical synthesis methods, it requires many procedures for exposing the selective ZnO seed surface.

On the other hand, hydrothermal synthesis has been considered as one of the promising methods for growing 1D ZnO nanostructures because it allows relatively low temperature (75–90 °C) and scalable manufacturing process [14–16]. Especially, this process is highly practical for various applications including field-effect transistors, solar cells, UV PDs, and piezoelectric devices [17–19]. By emerging the ZnO seed layer-coated substrates into aqueous solution, the ZnO NRAs are grown selectively on the seed layer, which enables to fabricate various device structures for specific device applications [20, 21]. In this letter, we demonstrated a facile fabrication and optimization of ZnO NRA-based MSM UV PDs by a hydrothermal growth. By the selective deposition of ZnO thin film using a shadow mask, the active channel for PDs was directly formed for the device fabrication. As compared with previous works of vertically aligned ZnO NRA-based UV PDs, this is a relatively convenient and controllable fabrication approach.

## Methods

**Fig. 1:**
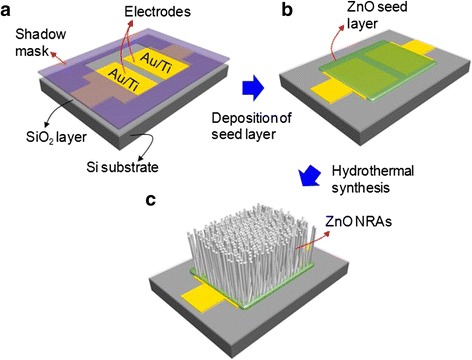
Schematic diagram. Fabrication of ZnO NRA-based MSM UV PDs: **a** selective exposure between two electrodes by a shadow mask, **b** deposition of the ZnO seed layer, and **c** hydrothermal growth of ZnO NRAs on the ZnO seed layer

Figure 1 displays the schematic diagram for the fabrication procedure of ZnO NRA-based MSM UV PDs: (a) selective exposure between two electrodes by a shadow mask, (b) deposition of the ZnO seed layer, and (c) hydrothermal growth of ZnO NRAs on the ZnO seed layer. First, we patterned Ti/Au electrodes on the SiO_2_-coated Si substrate. The Ti (5 nm) and Au (300 nm) films were deposited by e-beam evaporation, and the patterns were transferred by conventional photolithography and lift-off processes. After that, the sample was covered by the shadow mask during the radio frequency (RF) magnetron sputtering process. For the deposition of 50-nm-thick ZnO film, the RF magnetron sputtering was carried out at 6 mTorr of process pressure and 100 W of RF power under Ar environment. As depicted in Fig. 1a, the opened region in the shadow mask could be covered manually between the patterned electrodes under microscope observation. Then, to grow the ZnO NRAs, the ZnO seed layer-coated electrodes were immersed into the aqueous growth solution. Here, the growth solution was prepared by dissolving zinc nitrate hexahydrate and hexamethylenetetramine with equimolar concentrations (5–50 mM) in 200 mL of de-ionized (DI) water at room temperature. For obtaining a homogeneous solution, it was stirred with a magnetic bar for 2 h. After immersing the sample, it was placed into an oven at 90 °C for 5 h. Finally, the sample was carefully pulled out and dried by flowing DI water. To characterize the morphological and crystal properties, a field-emission scanning electron microscope (FE-SEM) (LEO SUPRA 55, Carl Zeiss, Germany) and an X-ray diffractometer (XRD) (Mac Science, M18XHF-SRA) were utilized. For photoresponse characteristics of the ZnO NRA-based UV PDs, the photocurrent was measured by using a semiconductor characterization system (Keithley 4200) under 2.8 mW/cm^2^ of illumination using a 365-nm UV light-emitting diode.

## Results and Discussion

**Fig. 2:**
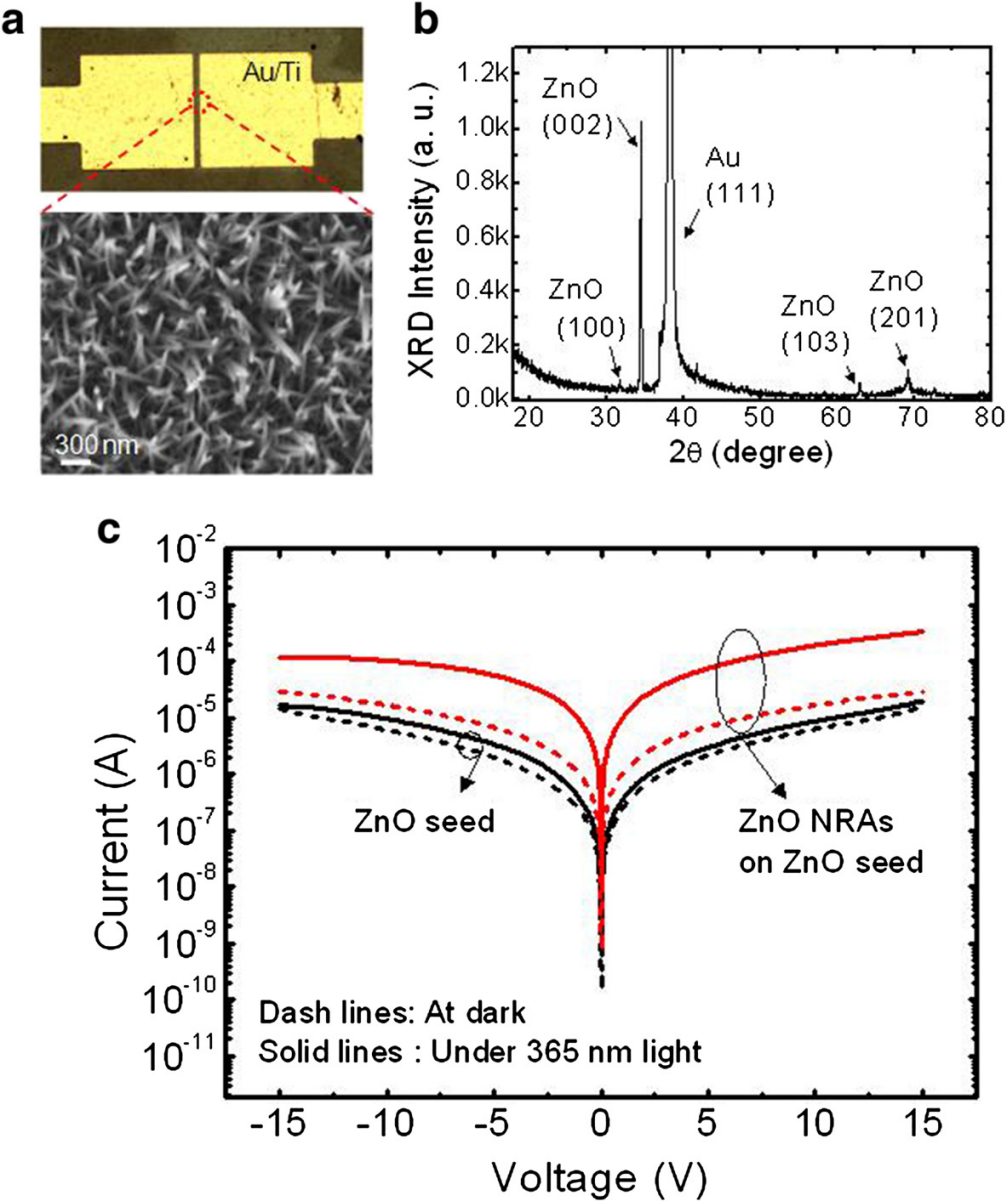
FE-SEM, XRD, and *I*-*V* curves. **a** Photographic and FE-SEM image of the ZnO NRAs between Au/Ti electrodes, **b** corresponding XRD patterns, and **c** measured *I*-*V* curves of the ZnO seed between the electrodes with and without ZnO NRAs at dark and under illumination of 365 nm light

Figure 2a shows the photographic and FE-SEM images of the vertically aligned ZnO NRAs on the gap between the Au/Ti electrodes. For hydrothermal synthesis, the growth solution was prepared with 25 mM of concentration. The Au/Ti electrodes were connected by the selective patterned ZnO thin film which acts as a seed layer for growing ZnO nanostructures. Typically, the ZnO nuclei are formed at the surface of seed layer, and the Zn(OH)_2_ nanorods grow in a perpendicular direction with reaction of hydroxide ions and zinc ions. After dehydration in air, Zn(OH)_2_ is naturally transformed into ZnO. As shown in the top-view FE-SEM image, the ZnO nanorods were densely distributed between the two electrodes.

Figure 2b shows the XRD patterns of ZnO NRAs in the device structure. According to the standard JCPDS card no. 89-1397, the ZnO XRD peaks are in good agreement with a hexagonal wurtzite crystal structure. Among the ZnO XRD peaks, the dominant (002) peak was observed at 2*θ* = 34.4°, indicating that the ZnO NRAs were grown and crystallized along the direction of the *c*-axis in the crystal structure. From the metal electrodes, the Au (111) XRD peak also appeared. To examine the photocurrent of the ZnO NRAs in this structure, the measured current-voltage (*I*-*V*) curves were compared, as shown in Fig. 2c, for the same channel width (i.e., gap distance between two electrodes) of 10 μm. Without ZnO NRAs, the ZnO seed layer between the two electrodes did not sufficiently generate a photocurrent. In contrast, the ZnO NRA-based PD exhibited a large photocurrent under the light illumination of 365 nm. In this condition, the device exhibited an on-off ratio of 25.3 at 5 V of bias voltage and a high photocurrent of 1.91 × 10^−4^ A at 10 V. As well-known in previous works [22], the photocurrent is attributed to the photogenerated electron-hole pairs in ZnO NRAs. Among them, the holes intend to be captured with trap levels at the surface of ZnO NRAs, and thereby the electrodes mainly contribute to enhancing the photocurrent. Here, the concentration of growth solution is closely related with the morphology of ZnO NRAs because it determines their size and height.

**Fig. 3:**
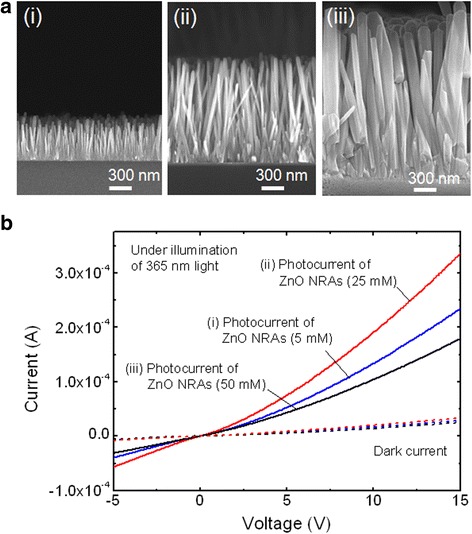
Morphology-dependent device characteristics of PD. **a** Cross-sectional views of FE-SEM images of the grown ZnO NRAs at different solution concentrations: (*i*) 5 mM, (*ii*) 25 mM, and (*iii*) 50 mM and **b** measured *I*-*V* curves of the corresponding samples at dark and under light illumination of 365 nm

In order to investigate the morphology-dependent device characteristics of PDs, we prepared the ZnO NRAs grown at different solution concentrations. Figure 3a shows the cross-sectional views of FE-SEM images for the ZnO NRAs grown at (i) 5 mM, (ii) 25 mM, and (iii) 50 mM. As the solution concentration increased, the diameter and height of ZnO NRAs increased gradually because more Zn ions were diffused to the ZnO nuclei, thus forming ZnO nanorods. At 5 and 25 mM, the diameters/heights of ZnO nanorods were ~30/573 nm and ~45/1350 nm, respectively. However, the diameter of ZnO nanorods was largely increased at 50 mM while the height was relatively less increased. This result can be explained by the fact that the excessive concentration of growth solution leads to a high growth rate of facets due to the induced isotropic growth of ZnO nanorods [23]. As shown in Fig. 3b, the measured photocurrents of the corresponding samples are compared under the same illumination condition with a fixed channel width of 10 μm. At 25 mM, the device exhibited the highest photocurrent. At 10 V of bias voltage, the photocurrents of 1.34 × 10^−4^, 1.91 × 10^−4^, and 1.04 × 10^−4^ A were obtained at solution concentrations of 5, 25, and 50 mM, respectively. As observed in the FE-SEM images of Fig. 3a, the surface area of ZnO NRAs is closely associated with the amount of generated photocurrent. The large size of ZnO nanorods at 50 mM reduces the surface area, which mainly results in the degradation of photocurrent due to the relatively low generation of electron-hole pairs. Also, the somewhat degraded crystallinity of large-sized ZnO NRAs could influence the reduction of photocurrent [24].

**Fig. 4:**
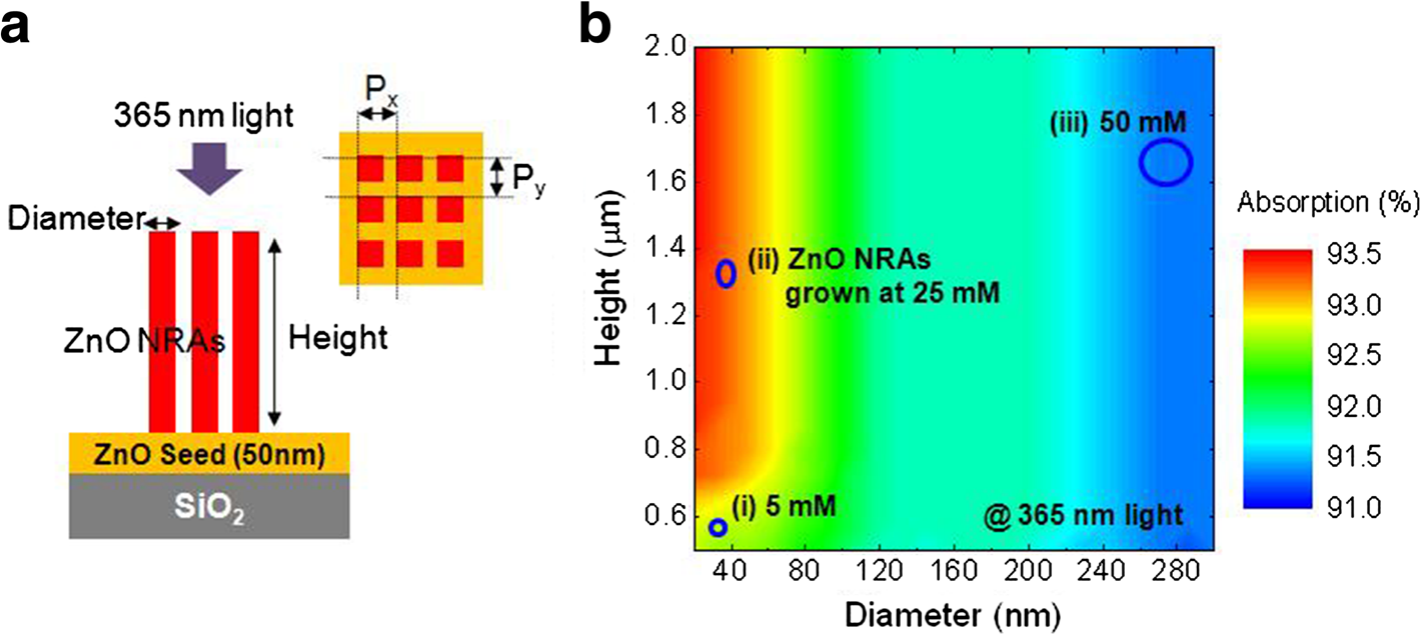
Absorption properties of ZnO NRAs. **a** Schematic diagram of ZnO NRAs for RCWA calculation and **b** color-coded absorption map as functions of diameter/height of the ZnO NRAs

Figure 4a shows the schematic diagram for investigating absorption properties of various ZnO NRAs using a rigorous coupled wave analysis (RCWA) simulation. For approximation, we assumed that the vertical ZnO nanorods are periodically aligned on the ZnO seed-coated SiO_2_ insulation layer, where *P*_*x*_ and *P*_*y*_ are the periods along *x* and *y* directions. At 365 nm of incident light, the complex refractive index of ZnO nanorods is considered to be 2.1 + i0.5 [25]. To set the distribution of ZnO NRAs with diameter (*W*) and height (*H*), the density (*D*) is defined by *W*^2^*/P*_*x*_*P*_*y*_. Figure 4b displays the color-coded absorption map as functions of diameter/height of the ZnO NRAs. Here, *D* is fixed to 0.7 and *P*_*x*_ equals *P*_*y*_. As *W* decreased or *H* increased, the absorption gradually increased from 91 to 93.5 %. For three different ZnO NRAs grown at (i) 5 mM, (ii) 25 mM, and (iii) 50 mM, the corresponding absorptions are marked in the map regarding their morphological properties by the SEM images. The ZnO NRAs grown at 25 mM exhibited a relatively high absorption of 93.4 % owing to multiple scattering of small-size and long ZnO nanorods [26]. In contrast, the ZnO NRAs grown at 5 and 50 mM have the lower absorptions of 92.8 and 91.3 %, respectively. This absorption trend of ZnO NRAs well agrees with the device characteristics of PD in Fig. 3b.

**Fig. 5:**
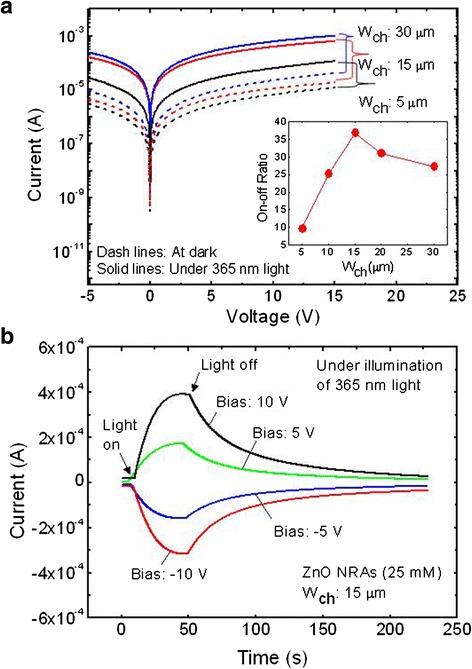
Influence of *W*_ch_ and photoresponse characteristics. **a** Measured *I*-*V* curves of the ZnO NRA-based PDs grown at 25 mM with different *W*_ch_ of 5, 15, and 30 μm and **b** photoresponse characteristics of the ZnO NRA-based PDs grown at 25 mM with *W*_ch_ of 15 μm under on/off switching of light illumination. The *inset* of **a** also shows the photocurrent on-off ratio with different *W*_ch_ from 5 to 30 μm

Additionally, the performance of ZnO NRA-based PDs is influenced by the channel width (*W*_ch_). Figure 5a shows the measured *I*-*V* curves of the ZnO NRA-based PDs grown at 25 mM with different *W*_ch_ of 5, 15, and 30 μm. When *W*_ch_ increased, the photocurrent also increased because the area of active region for absorbing the UV light and generating the photocurrent was increased. However, the dark current increased with the increase of *W*_ch_, which is a negative factor for the photocurrent on-off ratio. In fact, this dark current is mainly caused by the leakage of thermal noise from ZnO NRAs [27]. Indeed, this dark current originated from the thermally excited carriers in the active channel [28]. At room temperature, the thermal energy would excite the electrons from trap levels of ZnO NRAs to the conduction band and they are driven by an electrical potential. As *W*_ch_ increased, therefore, the dark current was enlarged by increasing the thermally excited carriers. This phenomenon was observed in the previous study of the channel length effect on dark current [29]. However, the photocurrent also increases with the increase of *W*_ch_ because the increased active area generates more electron-hole pairs. Therefore, *W*_ch_ was in a trade-off relationship with the photocurrent on-off ratio. As shown in the inset of Fig. 4a, the photocurrent on-off ratio was varied by changing the *W*_ch_. For *W*_ch_ = 15 μm, a high photocurrent on-off ratio of 37.4 was observed at 5 V of bias voltage. Above 20 μm, the photocurrent on-off ratio was gradually decreased because the dark current was more increased than the photocurrent with increase of the area of active region. Figure 5b shows the photoresponse characteristics of the ZnO NRA-based PDs grown at 25 mM with *W*_ch_ = 15 μm under on/off switching of light illumination. For positive and negative bias voltages, the symmetric photoresponse curves were clearly observed due to the structural property of the MSM device. Also, the device exhibited a slow decay process in typical transient curves of ZnO PDs. In fact, the re-adsorption of oxygen and water molecules occurs at ZnO, which interrupts electron-hole pair recombination. It is well known that the transient photocurrent in the ZnO material is attributed to the slow adsorption process [30, 31]. Prior to UV illumination, ambient oxygen molecules are adsorbed into the surface of ZnO nanorods and captured with pre-existing electrons in ZnO nanorods, which ionizes the adsorbed oxygen to O^−2^. After UV illumination, these ions are combined with photogenerated holes and the oxygen is desorbed at the surface of ZnO nanorods. This oxygen-related hole trap state mainly causes long recombination time, thus leading to the slow photoresponse [32, 33]. At 10 V, the proper photoresponse property was achieved with a reset time (i.e., current recover to e^−1^) of 26.1 s with a high photocurrent of 3.93 × 10^−4^ A.

## Conclusions

The ZnO NRA-based MSM PDs were facilely fabricated by the shadow mask technique and hydrothermal process. The selective deposition of ZnO seed layer offered a convenient and controllable fabrication method. For the optimization, the ZnO NRA-based PDs were prepared and characterized by changing the growth condition of ZnO nanorods and the *W*_ch_. For 15 μm of *W*_ch_, the ZnO NRAs grown at 25 mM exhibited a relatively high photocurrent on-off ratio of 37.4 in typical transient characteristics. These results can provide a deep insight into the simple fabrication for practical and efficient UV PD applications.

## References

[1] Chey CO, Liu X, Alnoor H, Nur O, Willande M (2014). Fast piezoresistive sensor and UV photodetector based on Mn-doped ZnO nanorods. Phys. Status Solidi RRL.

[2] Tsai DS, Lin CA, Lien WC, Chang HC, Wang YL, He H (2011). Ultra-high-responsivity broadband detection of Si metal–semiconductor–metal Schottky photodetectors improved by ZnO nanorod arrays. ACS Nano..

[3] Ko YH, Nagaraju G, Yu JS (2015). Wire-shaped ultraviolet photodetectors based on a nanostructured NiO/ZnO coaxial p–n heterojunction via thermal oxidation and hydrothermal growth processes. Nanoscale.

[4] Bai S, Wu W, Qin Y, Cui N, Bayerl DJ, Wang X (2011). High-performance integrated ZnO nanowire UV sensors on rigid and flexible substrates. Adv. Funct. Mater..

[5] Guo L, Zhang H, Zhao D, Li B, Zhang Z, Jiang M, Shen D (2012). High responsivity ZnO nanowires based UV detector fabricated by the dielectrophoresis method. Sen. Actuator B.

[6] Hassan JJ, Mahdi MA, Kasim SJ, Ahmed NM, Hassan HA, Hassan A (2012). High sensitivity and fast response and recovery times in a ZnO nanorod array/p-Si self-powered ultraviolet detector. Appl. Phys. Lett..

[7] Liu Y, Yang Q, Zhang Y, Yang Z, Wang ZL (2012). Nanowire piezo-phototronic photodetector: theory and experimental design. Adv. Mater..

[8] Wang X, Liao M, Zhong Y, Zheng JY, Tian W, Zhai T, Zhi C, Ma Y, Yao J, Bando Y, Golberg D (2012). ZnO hollow spheres with double-yolk egg structure for high-performance photocatalysts and photodetectors. Adv. Mater..

[9] Wang X, Tian W, Liao M, Bando Y, Golberg D (2014). Recent advances in solution-processed inorganic nanofilm photodetectors. Chem. Soc. Rev..

[10] Liu N, Fang G, Zeng W, Zhou H, Cheng F, Zheng Q, Yuan L, Zou X, Zhao X (2010). Direct growth of lateral ZnO nanorod UV photodetectors with Schottky contact by a single-step hydrothermal reaction. Appl. Mater. Interface.

[11] Ji LW, Peng SM, Su YK, Young SJ, Wu CZ, Cheng WB (2009). Ultraviolet photodetectors based on selectively grown ZnO nanorod arrays. Appl. Phys. Lett..

[12] Chen HY, Liu KW, Chen X, Zhang ZZ, Fan MM, Jiang MM, Xie XH, Zhao HF, Shen DZ (2014). Nanowire realization of a self-powered ZnO MSM UV photodetector with high responsivity using an asymmetric pair of Au electrodes. J. Mater. Chem. C.

[13] Liu B, Wang Z, Dong Y, Zhu Y, Gong Y, Ran S, Liu Z, Jing X, Zhong X, Chen D, Shen G (2012). ZnO-nanoparticle-assembled cloth for flexible photodetectors and recyclable photocatalysts. Mater. Chem.

[14] Guo M, Diao P, Cai S (2005). Hydrothermal growth of perpendicularly oriented ZnO nanorod array film and its photoelectrochemical properties. Appl. Surf. Sci..

[15] Xu S, Lao C, Weintraub B, Wang ZL (2008). Density-controlled growth of aligned ZnO nanowire arrays by seedless chemical approach on smooth surfaces. J. Mater. Res..

[16] Ko YH, Kim S, Yu JS (2013). Drop-cast and dye-sensitized ZnO nanorod-based visible-light photodetectors. Phys. Status Solidi RRL.

[17] Chen T, Liu SY, Xie Q, Jiang YL, Ru GP, Liu R, Qu XP (2010). Patterned ZnO nanorods network transistor fabricated by low-temperature hydrothermal process. Microelect. Eng..

[18] Zhou H, Fang G, Liu N, Zhao Z (2011). Ultraviolet photodetectors based on ZnO nanorods-seed layer effect and metal oxide modifying layer effect. Nanoscale Res. Lett..

[19] Ko YH, Yu JS (2013). Preparation of ZnO nanorods on cellulose fiber paper and their charge-generating application for waste paper recycling. Phys. Status Solidi RRL.

[20] Ko SH, Lee D, Hotz N, Yeo J, Hong S, Nam KH, Grigoropoulos CP (2012). Digital selective growth of ZnO nanowire arrays from inkjet-printed nanoparticle seeds on a flexible substrate. Langmuir.

[21] Xu S, Wei Y, Kirkham M, Liu J, Mai W, Davidovic D, Snyder RL, Wang ZL (2008). Patterned growth of vertically aligned ZnO nanowire arrays on inorganic substrates at low temperature without catalyst. J. Am. Chem. Soc..

[22] Hasan K, Alvi NH, Lu J, Nur O, Willander M (2011). Single nanowire-based UV photodetectors for fast switching. Nanoscale Res. Lett..

[23] Baruah S, Dutta J (2009). Hydrothermal growth of ZnO nanostructures. Sci. Technol. Adv. Mater..

[24] Ko YH, Yu JS (2010). Structural and antireflective properties of ZnO nanorods synthesized using the sputtered ZnO seed layer. J. Nanosci. Nanotechnol..

[25] Gayen RN, Bhar R, Pal AK (2010). Synthesis and characterization of vertically aligned ZnO nanorods with controlled aspect ratio. Indian J. Pure Appl. Physc..

[26] Baek SH, Noh BY, Park LK, Kim JH (2012). Fabrication and characterization of silicon wire solar cells having ZnO nanorod antireflection coating on Al-doped ZnO seed layer. Nanoscale Res. Lett..

[27] Xie HQ, Zeng Y, Yan YH, Zhang GL, Wang TH (2009). Characterization of responsivity, sensitivity and spectral response in thin film SOI photo-BJMOS-FET compatible with CMOS. Technology World Acad. Sci. Eng. Technol..

[28] Sang D, Li H, Cheng S, Wang Q, Liu J, Wang Q, Wang S, Han C, Chen K, Pan Y (2015). Ultraviolet photoelectrical properties of a n-ZnO nanorods/p-diamond heterojunction. RSC Adv..

[29] Xie H, Zeng Y, Zeng J, Wang T (2011). Analysis and simulation of lateral PIN photodiode gated by transparent electrode fabricated on fully-depleted SOI film. J. Cent. South Univ. Technol..

[30] Dhara S, Giri PK (2011). Enhanced UV photosensitivity from rapid thermal annealed vertically aligned ZnO nanowires. Nanoscale Res. Lett..

[31] Bao J, Shalish I, Su Z, Gurwitz R, Capasso F, Wang X, Ren Z (2011). Photoinduced oxygen release and persistent photoconductivity in ZnO nanowires. Nanoscale Res. Lett..

[32] Studenikin SA, Golego N, Cocivera M (2000). Carrier mobility and density contributions to photoconductivity transients in polycrystalline ZnO films. J. Appl. Phys..

[33] Li QH, Gao T, Wang YG, Wang TH (2005). Adsorption and desorption of oxygen probed from ZnO nanowire films by photocurrent measurements. Appl. Physc. Lett..

